# Study of Toxic Elements in River Water and Wetland Using Water Hyacinth (*Eichhornia crassipes)* as Pollution Monitor

**DOI:** 10.1002/gch2.201800087

**Published:** 2019-01-30

**Authors:** Alok Srivastava, Vikas Chahar, Vishal Sharma, Kollola K. Swain, Friedrich Hoyler, Ganti S. Murthy, Ulrich W. Scherer, Hildegard Rupp, Friedhart Knolle, Miyuki Maekawa, Ewald Schnug

**Affiliations:** ^1^ Chemistry Department Panjab University Chandigarh 160014 India; ^2^ Analytical Chemistry Division Bhabha Atomic Research Centre Mumbai 400085 India; ^3^ Aachen University of Applied Sciences Heinrich‐Mussmann‐Str. 1 D‐52428 Juelich Germany; ^4^ Biological and Ecological Engineering Oregon State University Corvallis OR 97331 USA; ^5^ Hochschule Mannheim University of Applied Sciences Institute of Physical Chemistry and Radiochemistry Paul‐Wittsack‐Str. 10 D‐68163 Mannheim Germany; ^6^ Institute of Geosystems and Bioindication Technical University of Braunschweig Langer Kamp 19c D‐38106 Braunschweig Germany; ^7^ UNESCO Global Geopark Harz. Braunschweiger Land Ostfalen Network Grummetwiese 16 D‐38640 Goslar Germany; ^8^ Department of Life Sciences Technical University of Braunschweig Pockelsstr. 14 D‐38106 Braunschweig Germany

**Keywords:** ICP‐MS, rhizofiltration, toxic elements, water hyacinth, water monitoring

## Abstract

The concentration of toxic elements present in surface water of Sutlej River and Harike wetland besides *Eichhornia crassipes*, commonly known as water hyacinth, is estimated employing inductively coupled plasma mass spectrometry (ICP‐MS). Toxic elements such as cadmium (Cd), chromium (Cr), copper (Cu), manganese (Mn), nickel (Ni), lead (Pb), uranium (U), and zinc (Zn) are identified in the river as well as in Harike wetland catchment. Accumulation of elements in different parts of the water hyacinth plant is observed with the roots exhibiting maximum affinity followed by stem and then leaves. The removal efficacy of pollutants by water hyacinth is estimated using bioconcentration factor (BCF) index. It is found to be different for different elements, with Mn showing the highest and U the lowest magnitude. The study carried out in the present work indicates that rhizofiltration could play an important role in controlling pollutant load.

## 1 Introduction

The study of toxic metal pollution is of utmost importance when discussing key environmental issues. Metals are nonbiodegradable and are placed second only to pesticides in terms of their impact on biodiversity. They too can play havoc with biodiversity if not checked in time.

The Sutlej River is the longest of the five rivers that flow through the historic crossroads region of Punjab in northern India and Pakistan. It is located north of the Vindhya Range, south of the Hindu Kush segment of the Himalayas. It is the easternmost tributary of the Indus River. The Indus River system belongs to the most fragmented river systems in the world after more than 150 years of barrage construction and abstraction of water to feed irrigated agriculture. The waters of the Sutlej are allocated to India under the Indus Water Treaty between India and Pakistan, and are mostly diverted to irrigation canals in India. There are several major hydroelectric projects on the river. On the other hand Beas River originates in western part of Himalayas in central Himachal Pradesh and after passing through the Kulu and Kangra Valley enters Punjab State meeting Sutlej at Harike.

### 1.1 Harike Wetland

The barrage at Harike Pattan, situated at the confluence of the rivers Sutlej and Beas, was constructed in 1952. The water is pushed further into two major canals one of which irrigates South West Punjab region from where uranium (U) toxicity has been reported.^1^, ^2^ The toxic elements that are present in the water of Beas and Sutlej find their way into the crops which may be one of the causes of health related problems in the region.^3^, ^4^ The river downstream of Harike barrage is virtually dry, only connected to the rest of the river system for a few weeks during the monsoon floods. National River Conservation Directorate, Ministry of Environment and Forests and Government of India monitor the regional river water quality since 1996.

Braulik et al.^5^ who examined the causes of the range decline of the Indus River dolphin (*Platanista gangetica minor*), noted that dam construction has dramatically increased habitat fragmentation and degradation in fresh water ecosystems. Dams, barrages, irrigation canals, siltation, changes in river course, artificial embankments, sand mining, riparian agriculture, and land‐use changes are among major threats for once reportedly abundant and now endangered riverine taxa, such as river dolphin (*P. gangetica*), smooth‐coated otter (*Lutrogale perspicillata*), gharial (*Gavialis gangeticus*), and mugger crocodile (*Crocodylus palustris*), in addition to numerous waterfowl and fish species. The gharial population of the Indus River system is presumed to be extinct.^6^ The range decline of the Indus River dolphin accounted for 80% by the early 1990s. It was extirpated in most river fragments, but persists in six small subpopulations, one of them in Beas River, being genetically isolated since decades.^5^

The artificial reservoir upstream Harike barrage became a habitat for water fowls and was declared as nature reserve in 1982 because of its rich biodiversity. Since 1990 Harike wetland is protected as a Ramsar Site by the International Union for Conservation of Nature (IUCN) and in 1992 the Indian government declared it a Bird Sanctuary to maintain the breeding ground for a large variety of migratory and domiciled birds.

Nevertheless pollution, eutrophication, infestation with exotic weed, in particular water hyacinth (*Eichhornia crassipes* (*Mart*.) *Solms, Pontederiaceae*), and siltation caused degradation and shrinkage of the wetland. Evaluation of satellite data revealed, that the wetland area decreased approximately by 30% in size in the period from 1990 to 2003.^7^ Bathymetric surveys of Harike Lake in 2010 indicated that the wetland had lost 83% of its water holding capacity over the last 54 years as a result of rapid siltation reducing water‐bearing space and permeability in the vegetation complex. Continued discharge of pollutants from upstream townships brought into Harike by rivers Sutlej and Beas have led to extensive proliferation by aquatic plant invasives. The open water expanse has also significantly reduced from 4100 ha in 1952 to 2800 ha at present. Degradation of wetlands puts the significant hydrological services, as well as biodiversity habitats at risk.^8^, ^9^

Moza and Mishra^10^ who implemented investigations on changes of biodiversity of Harike wetland, note the absence of larvae of Trichopteran (caddisflies), Neuropteran (lacewings) and *Ephemerella* sp. (mayflies) among insects as indicators for cleaner environments that had been found in former studies carried out by Bath and Kaur^11^ between 1990 and 1993. The exotic catfish *Clarias gariepinus* occurs since 2002,^10^ which raised concerns due to its negative impacts on native fish fauna through predation, as well as the fact that it can hybridize with the morphologically similar indigenous catfish *Clarias batrachus*, *C. gariepinus*is probably still reared in aquaculture though being banned by the Indian Ministry of Agriculture in 1997. The status of the genus *Clarias* at Harike remains unclear.

The rapid growth and proliferation rate of water hyacinth is a global concern since it poses serious environmental and conservation problems having socioeconomic repercussions.^12^ The weed growth prevents surface aeration and penetration of sunlight, which in turn affects the dissolved oxygen content in the lake and consequently influencing the biodiversity of the lakes flora and fauna.^13^

### 1.2 Environmental Monitoring

The river Sutlej is known to carry the major effluent load of Ludhiana, one of the most polluted industrial cities of India where industries are primarily engaged in the manufacture of machine parts, chemicals, hosiery, leather, dyeing, electroplating, etc.^14^, ^15^, ^16^, ^17^, ^18^ The heavy metals cadmium (Cd), lead (Pb), zinc (Zn), copper (Cu), nickel (Ni), and chromium (Cr) have already been identified in the effluents of the industries located in and around Ludhiana. The river Beas though not as polluted as Sutlej too is known to carry somewhat significant domestic and industrial effluents.

Therefore, the regulatory authorities and others have been monitoring the water quality parameters of the Harike wetland and their influent streams. Brraich and Jangu^19^ have reported the pollution of Harike wetland by metallic pollutants by carrying out measurements on the concentration of metals like aluminum, chromium, nickel, iron, and lead in the scales of the *Cyprinus carpio* (Linnaeus) commonly known as the common Carp using energy dispersive X‐ray microanalysis. Kaur et al.^20^ studied mutagenic and genotoxic effects of surface water samples from Harike wetland using the histidine reversion point mutation assay in *Salmonella typhimurium* (TA98) strain and plasmid nicking assay using pBR322, as well as toxic metals concentrations. It was observed that although the water samples of all the sites demonstrated mutagenic as well as genotoxic activity, the effect was quite significant with the water samples from sites containing water from river Sutlej. The authors postulate that high level of pollution due to industrial effluents and agricultural run‐off at these sites may engender the genotoxicity and mutagenicity of drinking water.

The outcomes of a comprehensive research study^21^, ^22^, ^23^, ^24^, ^25^ undertaken to understand the factors that influence the water quality of river Sutlej by its catchment indicate further effect on the ecosystem of Harike wetland. A specific water quality index for ecological use has been developed to evaluate the water quality of river Sutlej. The selected parameters grouped into three subindices categories (general parameters, toxic metals, pesticides, and other toxic organic compounds). Most of the flow in the river upstream of Harike wetland was contributed by the Buddha drain and the East Bein River subwatershed. Further, storm water component in the water drained by these two subwatersheds is almost insignificant. Most of the water was urban sewage and industrial effluents. Biodegradable organic matter, nutrients, and toxic elements and toxic organic substances were the key pollutants in these waters.

Water hyacinth has been found to be an excellent bio‐monitor for the study of toxic metals.^26^, ^27^, ^28^, ^29^, ^30^, ^31^, ^32^, ^33^, ^34^ Its efficacy as a biomonitoring tool has been demonstrated in the study of toxic metal concentration in the river Asa of Nigeria^29^ and the river Nile of Egypt^30^ using neutron activation analysis besides the tropical river Morichal Largo River of Venezuela,^31^ Lerma River of Mexico^32^ and the Mukuvisi and Manyame Rivers of Zimbabwe.^33^

In the present work, efficacy of water hyacinth as pollution monitor of toxic elements present in Sutlej River and Harike wetland catchment is reported by determining the concentration of Cd, Cr, Cu, Mn, Ni, Pb, U, and Zn in water as well as water hyacinth collected from different locations along an 80 km stretch was using inductively coupled plasma mass spectrometry (ICP‐MS) technique.

## 2 Results and Discussions

Eight toxic elements namely Cd, Cr, Cu, Mn, Ni, Pb, U, and Zn which are known to adversely affect aquatic plant and animal species were identified in Sutlej River and the catchment area of Harike wetland. The concentrations of above stated toxic elements in leaves, shoots, and roots in water hyacinth on dry weight basis along with their concentrations in surface water determined using ICP‐MS technique are shown in **Table** **1**. The mean values in Table 1 are based on triplicate measurements. The concentration of toxic elements obtained in the present work in surface water is found to be well within the permissible limit recommended by regulatory authorities^35^, ^36^ for drinking purposes. The aforementioned table also shows the bioconcentration factor (BCF) which was evaluated using the ratio of the concentration of the elements of interest in the root of the plant and the concentration of the water from where the plant was collected.

**Table 1:** Mean concentrations of toxic elements in different parts of water hyacinth (mg kg^−1^) and surface water (µg L^−1^) along with bio‐concentration factor (BCF)

Sample		Cd	Cr	Cu	Mn	Ni	Pb	U	Zn
WH1 (Sutlej)	Leaf	0.07	8.8	20.9	125	4.9	1.5	0.14	64.8
	Stem	0.06	13.6	16.7	269	7.9	1.7	0.18	81.1
	Root	2.6	44.4	50.3	4755	47.0	20.4	10.8	102.1
Water (µg L^−1^)		ND	0.15	2.10	0.16	0.62	0.28	1.15	3.73
BCF * (10^3^)		–	296.3	23.9	29 719	75.8	72.9	9.4	27.4
WH2 (Sutlej)	Leaf	0.04	7.7	23.9	212	4.4	2.1	0.15	33.9
	Stem	0.03	7.5	20.6	197	3.6	1.4	0.09	40.0
	Root	1.3	23.3	47.8	5310	41.5	10.9	7.04	113.8
Water (µg L^−1^)		ND	0.17	1.11	0.18	1.30	0.16	1.24	1.61
BCF * (10^3^)		–	137.1	43.1	29 501	31.9	68.4	5.7	70.7
WH3 (Sutlej)	Leaf	0.04	2.3	19.5	194	2.1	1.6	0.11	71.8
	Stem	0.04	2.6	24.3	470	4.6	1.6	0.22	194.1
	Root	0.53	53.1	33.7	2728	38.4	15.3	7.96	124.3
Water (µg L^−1^)		ND	0.37	2.07	0.21	1.57	0.36	0.16	3.00
BCF * (10^3^)		–	143.5	16.3	12 992	24.5	42.4	49.7	41.4
WH4 (Harike catchment)	Leaf	0.02	3.4	9.4	236	2.6	0.7	0.08	29.9
	Stem	0.03	3.8	17.0	206	2.4	0.9	0.04	61.4
	Root	0.75	93.6	34.7	1735	30.1	9.0	1.98	134.7
Water (µg L^−1^)		ND	0.31	0.84	3.72	0.63	0.20	0.18	1.69
BCF * (10^3^)		–	301.9	41.3	466.5	47.8	45.0	11.0	79.7
WH5 (Harike catchment)	Leaf	0.07	3.6	23.8	514	2.3	1.7	0.08	173.3
	Stem	0.05	7.8	20.6	689	4.2	1.2	0.19	41.0
	Root	0.48	111	45.3	5414	40.2	14.4	4.61	98.3
Water (µg L^−1^)		ND	0.11	1.06	0.27	2.64	0.14	0.38	2.38
BCF * (10^3^)		–	1017	42.7	20 053	15.2	103.3	12.1	41.3
WH6 (Buddha Drain)	Leaf	0.04	7.4	13.4	100	10.4	1.8	0.17	92.4
	Stem	0.03	14.0	23.5	153	29.7	2.1	0.15	181.7
	Root	1.3	624	148	433	286	63.7	14.19	2003
Water (µg L^−1^)		ND	1.23	2.03	0.47	8.71	0.35	1.06	0.91
BCF * (10^3^)		–	507.6	73.3	921.8	32.8	182.1	13.4	2201
WHO permissible limits (water) ppb		5	50	50	100	20	10	15	5000
BIS permissible limits (water) ppb		3	50	50	30	20	10	–	15 000

BCF indicates bio‐concentration factor with respect to roots of water hyacinth. Instrumental RSD: within 5%.

The concentrations of toxic elements in the water hyacinth obtained from rivers located in different parts of the world as reported in literature^30^, ^32^, ^37^, ^38^, ^39^ are shown in **Table** **2**.

**Table 2:** Concentration (mg kg^−1^) range of studied elements in water hyacinth collected from Sutlej/Harike wetland besides in those reported from other tropical rivers of the world

Element (mg kg^−1^)	Sutlej River/Harike wetland (present work)	Nile River^30^	Lerma River^32^	Nile River^37^	Imbe River^39^	Almendares River^38^	Paraíba do Sul River^39^
Cd	0.5–1.3	–	–	–	–	–	–
Cr	23.3–624	8.32–66	1.0–17.3	–	4–10	18–281	4.0–24.0
Ni	30.1–286	–	1.2–10.8	5.65–10.8	1.77–2.11	<1.9–62	3.94–10.6
Pb	9.0–63.7	–	1.9–7.3	–	0.98–4.03	1.43–9.01	1.43–9.01
Zn	98.3–2003	30.5–1358	13.9–91.2	24.75–660	56–67	35 – 392	64–228
Cu	33.7–149	–	3.6–21.5	17.48–115	9.97–14	11–74	13.9–22.3
Mn	433–5414	–	130–1760	–	1580 – 3558	–	637–4883
U	1.9–14.2	6.3–48	–	–	–	–	–

It is observed that in general the level of toxic elements in water hyacinth obtained from Sutlej River/Harike wetland is considerably higher in comparison to those obtained from tropical rivers from other parts of the world. As a result of translocation the concentration of toxic elements in leaves and shoot are observed to be lower than root but nevertheless significant. The total concentration of toxic elements in water hyacinth becomes several orders higher than that observed in water: typical for phyto‐accumulation effects, a well‐known characteristic displayed by water hyacinths due to which they are exploited as rhizofilters.

The BCF reflects the affinity of a given biomonitor to specific pollutants. High BCF values reflect higher affinity. In the present study water hyacinth showed the highest BCF values for Mn > Cr > Zn > Pb > Cu > Ni > U as observed in Table 1.

As many aquatic species have generally long residence time in water bodies similar to water hyacinth it is quite likely that akin to it they too may be accumulating toxic elements thereby creating a significant cumulative load of toxicants on them. It has been reported^40^, ^41^, ^42^, ^43^ that aquatic species do tend to bioaccumulate metals over long periods of time and the bioaccumulation is dependent on nature and concentration of the dissolved metallic species besides different biotic and abiotic factors.

The low level of toxic elements in the water of Sutlej River and Harike catchment may be attributed to rhizofiltration by aquatic species besides mobilization of toxic elements in the riverine system which is generally dominated by suspended and bed sediments. It has been reported^44^, ^45^, ^46^, ^47^, ^48^, ^49^, ^50^ that suspended solids appear to increase the bioavailability of toxic metals to aquatic life by accumulating toxic metals and then transferring them from terrestrial to aquatic environment. Tiwana et al.^51^ presented on the status of Ramsar sites of Punjab namely Ropar, Kajli, and Harike have shown that siltation has become a major problem at the Ramsar sites. This gives further credence to the hypothesis that sediments may be playing a significant role in this case.

It would be important to carry out further study on mobilization of toxic elements by water and sediments besides different types of aquatic species like water hyacinth, duckweed, phytoplanktons, zooplanktons, fishes, etc. to get more meaningful picture about toxicity arising in wetlands due to chemical elements present in domestic and industrial effluents. Biomass management methods could be useful in this case to protect the ecosystems damaged by water hyacinths. The most purposeful way to protect the ecosystems of the rivers and the Ramsar site is just to massively reduce the input of toxic riverine emissions.

## 3 Conclusions

In conclusion it can be stated that the present work has been successful in demonstrating the presence of toxic elements at trace and ultratrace levels in Sutlej River and Harike wetland. The accumulation of toxic elements has been found to be significantly higher in the roots compared to stems and leaves. The range of concentration of toxic elements in water hyacinth collected from Sutlej River and the Harike wetland catchment was found to be considerably higher than those obtained from tropical rivers located in different parts of the world. The study carried out shows the important role rhizofiltration may be playing in controlling the pollutant load of Sutlej River and Harike wetland.

## 4 Experimental Section

**Figure 1:**
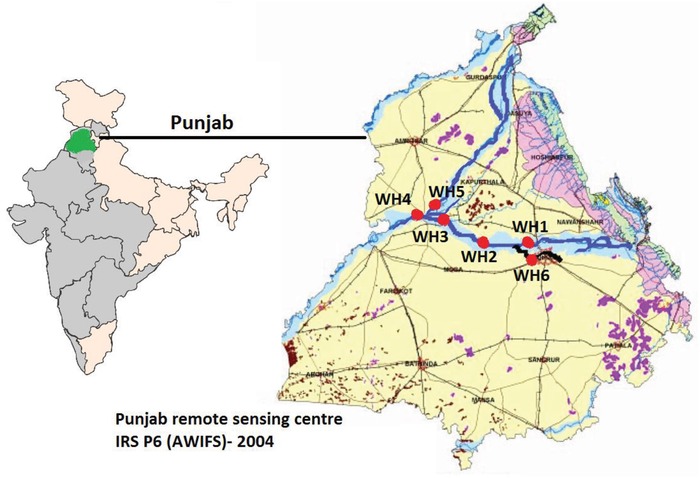
Locations of the sampling stations, from where surface water and water hyacinth were collected.

*Sample Collection*: The geographic location of the studied area in Punjab (India) lies between 30.910643 and 31.179414 N latitude and 75.880251 and 74.943581 E longitude. The river water samples were collected from different locations along the stretch of Sutlej from Phillaur to Harike wetland. In the field trip carried on 24.10.2016, in addition to river water samples, water hyacinth samples were also collected from locations where they were found. The information about the respective locations from where samples related to surface water and water hyacinth were collected is shown in **Figure** **1**.

The water samples were collected in polyethylene bottles which were soaked in 10% HNO_3_ (65% ultrapure, Himedia) for 24 h, washed twice with Millipore water and rinsed thrice by water to be sampled. The collection point chosen for the samples ranged between 10 and 15 m from the bank depending on the topography of the river. The water was collected from a depth ranging between 50 and 60 cm. Ultrapure nitric acid was used to preserve the water samples by bringing down the pH below 2. Water hyacinth samples were collected in clean polyethylene bags and washed with Millipore water thrice to remove the unwanted organic matter on the surface of root stem and leaves.

WH1, WH2, and WH3 depict locations from where surface water and water hyacinth samples of Sutlej River were collected whereas WH4 and WH5 depict locations from where surface water and water hyacinth of Harike wetland catchment were collected and WH6 is the location from where surface water and water hyacinth of Buddha drain which falls into Sutlej River was collected.

*Chemicals*: Nitric acid, HNO_3_ (supra, 69%, Carl Roth), H_2_O_2_ (puriss, 30%, Sigma‐Aldrich) were applied as received. Ultrapure water with conductivity of 0.054 µS cm^−1^ was supplied from a Merck‐Chemicals Milli‐Q Academic system. Single element aqueous standards of Ge (1000 µg mL^−1^) and U (997 µg mL^−1^) were provided from LHG Labs, Rh (1000 mg L^−1^) from Bernd Kraft and all other standard solutions (ROTI STAR for ICP, 1000 mg L^−1^) from Carl Roth.

Standard solutions for calibration curves containing the all elements which were measured were prepared from single element standard solutions. For ICP‐MS, an internal standard solution was prepared from Rh and Ge standard solution by dilution with 2% HNO_3_, which was prepared from 69% HNO_3_ and ultrapure water. The concentrations of each element are 1 mg L^−1^(Rh) and 10 mg L^−1^ (Ge). Rh was used as internal standard for low/middle resolution and Ge for high resolution.

*Sample Preparation (Water Samples)*: Water samples were filtered through 0.45 µm filter paper to remove insoluble particles if any. All the samples were concentrated by nearly ten times by evaporation method (by evaporating water in a hot oven at 60 °C) before analysis of toxic elements. For ICP‐MS measurement each 5 mL of samples were taken, HNO_3_ (50 µL) and H_2_O_2_ (50 µL) were added into the sample. The acidified samples were irradiated by UV light using a Metrohm 705 UV Digester for 2 h. After irradiation, the samples were filtrated using Rotilabo‐syringe filter (⌀ 0.45 µm, CME, Carl Roth). Each 400 µL of digested samples were filled up to 10 mL with 2% HNO_3_ after addition of internal standard solution (10 µL).

*Sample Preparation (Plant Samples)*: Root, stem and leaves were separated, chopped into fine pieces, which were then dried at 45 °C to remove the moisture content and then were pulverized to fine powder and stored in polyethylene packets for further processing. Each 5 g of samples were weighted and put into a Teflon vessel with HNO_3_ (6 mL) and H_2_O_2_ (1.5 mL). The sample was treated in a MARSXpress microwave oven (CEM) with the temperature program below: 1) heated from ambient temperature to 120 °C within 5 min., then kept for 2 min. 2) heated from 120 to 200 °C within 10 min., then kept for 15 min. 3) cooled to 50 °C within 30 min., then kept for 5 min. After cooling down to ambient temperature all contents were transferred into volumetric flask and filled up to 50 mL with ultrapure water. The sample was then treated through a filter paper (Sartorius Quantitative Grade 292, ⌀ 5–8 µm).

For the ICP‐measurements, samples were diluted as required before analysis.

*Instrumentation*: All measurements were performed using a Thermo Fisher Scientific Element XR (MS) Spectrometer which was equipped with an MC‐SEV‐ICP II‐V2 electron multiplier (MasCom), a Micro Mist concentric nebulizer (Glass Expansion) and a twister spray chamber with helix (Glass Expansion). The radiofrequency generator attached to the Instrument was operated at 1240 W power. The peristaltic pump attached to was adjusted so as to have a speed of 12 rpm. The sample flow was manually kept at 1.105 L min^−1^ and the auxiliary gas flow at 0.75 L min^−1^ whereas the coolant gas was regulated automatically at a speed of 16.0 L min^−1^. The torch position, lens parameters, and the nebulizer gas flow rate were optimized daily to obtain maximum sensitivity and held constant during the subsequent analytical work.

## Conflict of Interest

The authors declare no conflict of interest.
